# Increased susceptibility to kainate‐induced seizures in a mouse model of tuberous sclerosis complex: Importance of sex and circadian cycle

**DOI:** 10.1002/epi4.12955

**Published:** 2024-07-15

**Authors:** Mariana L. Pais, João Martins, Miguel Castelo‐Branco, Joana Gonçalves

**Affiliations:** ^1^ Coimbra Institute for Biomedical Imaging and Translational Research (CIBIT), R. Santa Comba University of Coimbra Coimbra Portugal; ^2^ Institute for Nuclear Sciences Applied to Health (ICNAS), R. Santa Comba University of Coimbra Coimbra Portugal; ^3^ Institute of Physiology, Faculty of Medicine University of Coimbra Coimbra Portugal

**Keywords:** autism spectrum disorder, biological sex, circadian cycle, epilepsy, tuberous sclerosis complex

## Abstract

**Objective:**

Comorbidity of epilepsy and autism in tuberous sclerosis complex 2 (TSC2) is very frequent, but the link between these conditions is still poorly understood. To study neurological problems related to autism, the scientific community has been using an animal model of TSC2, *Tsc2* ^*+/−*^ mice. However, it is still unknown whether this model has the propensity to exhibit increased seizure susceptibility. Further, the importance of sex and/or the circadian cycle in this biological process has never been addressed. This research aimed to determine whether male and female *Tsc2* ^*+/−*^ mice have altered seizure susceptibility at light and dark phases.

**Methods:**

We assessed seizure susceptibility and progression in a *Tsc2* ^*+/−*^ mouse model using the chemical convulsant kainic acid (KA), a potent agonist of the AMPA/kainate class of glutamate receptors. Both male and female animals at adult age were evaluated during non‐active and active periods. Seizure severity was determined by integrating individual scores per mouse according to a modified Racine scale. Locomotor behavior was monitored during control and after KA administration.

**Results:**

We found increased seizure susceptibility in *Tsc2* ^*+/−*^ mice with a significant influence of sex and circadian cycle on seizure onset, progression, and behavioral outcomes. While, compared to controls, *Tsc2* ^*+/−*^ males overall exhibited higher susceptibility independently of circadian cycle, *Tsc2* ^*+/−*^ females were more susceptible during the dark and post‐ovulatory phase. Interestingly, sexual dimorphisms related to KA susceptibility were always reported during light phase independently of the genetic background, with females being the most vulnerable.

**Significance:**

The enhanced susceptibility in the Tsc2 mouse model suggests that other neurological alterations, beside brain lesions, may be involved in seizure occurrence for TSC. Importantly, our work highlighted the importance of considering biological sex and circadian cycle for further studies of TSC‐related epilepsy research.

**Plain Language Summary:**

Tuberous sclerosis complex (TSC) is a rare genetic disorder. It causes brain lesions and is linked to epilepsy, intellectual disability, and autism. We wanted to investigate epilepsy in this model. We found that these mice have more induced seizures than control animals. Our results show that these mice can be used in future epilepsy research for this disorder. We also found that sex and time of day can influence the results. This must be considered in this type of research.

## Graphical abstract

Insights from the TSC2 mouse model: Common aspects and differences from the clinical manifestations. TSC is a rare genetic disorder caused heterozygous mutations in either the *Tsc1* or *Tsc2* genes, typically leading to serious neurologic comorbidities, such as severe epilepsy. Similar to the clinical condition, rodents with a heterozygous genotype in *Tsc2* display mTOR hyperactivation and autistic‐like features. Significantly, we showed that even though the TSC2 mouse model does not have spontaneous epileptic events or significant pathological brain abnormalities, *Tsc2* ^*+/−*^ animals are more vulnerable to KA‐induce seizures. Moreover, we further exposed a significant effect of sex and circadian cycle for seizure vulnerability. On one side, compared to controls, *Tsc2* ^*+/−*^ males exhibit a constant higher susceptibility for both circadian phases. On the other, *Tsc2* ^*+/−*^ female when compared with control animals were generally more vulnerable to KA‐induced seizures during the dark phase and for the post‐ovulatory stage of their estrous cycle. The figure was partly generated using Servier Medical Art, provided by Servier, licensed under a Creative Commons Attribution 3.0 unported license.

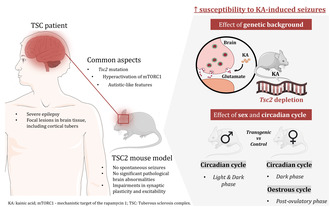

**Key points**

- *Tsc2* ^*+/−*^ animals exhibit higher susceptibility to KA‐induced seizures.
- Seizure susceptibility and behavior outcomes vary based on sex and circadian period.
- Sexual dimorphisms related to KA susceptibility exclusively occur during light phase independently animal's genetic background, with females being the most vulnerable.
- Female vulnerability depends on hormonal fluctuations and circadian cycle changes according to their genetic background.

## 1 INTRODUCTION

Tuberous sclerosis complex (TSC) is a rare genetic disorder that leads to serious neurologic comorbidities such as severe epilepsy, intellectual disability, and autistic features.^1^ Epilepsy is the most common neurological complication of TSC, and up to 80%–90% of individuals with TSC develop seizures at some point in their lifetime.^2^, ^3^ Despite the continuous development of new drugs, pharmacoresistance in patients with TSC is common and a matter of concern,^3^, ^4^ and further studies are needed to better characterize epilepsy in TSC.

This disease is caused by heterozygous mutations in either *Tsc1* or *Tsc2* genes, which usually function to suppress the mechanistic target of the rapamycin 1 (mTORC1) pathway.^3^, ^5^ In TSC, abnormal hyperactivation of mTORC1 promotes cell growth that contributes to tumor formation or focal lesions in brain tissue, including cortical tubers, which are often considered the foci of epileptic seizures.^2^, ^4^, ^6^, ^7^ However, it remains controversial whether epileptic episodes can be triggered within the tubers, as in rare cases, they can arise entirely remotely from these lesions.^8^, ^9^ Similar to clinical conditions, rodents with a heterozygous genotype in either *Tsc1* or *Tsc2* display mTOR hyperactivation but without significant pathological brain abnormalities,^8^ and therefore, can be used to study seizure occurrence in this condition without the presence of these lesions.

Interestingly, heterozygous TSC models show autism‐like features, such as social, cognitive, and behavioral deficits indicating that gross pathological lesions and loss of heterozygosity are not necessary to cause these neurological symptoms.^8^, ^10^, ^11^ Heterozygotic *Tsc2* mutations are the most prevalent and the most frequently associated with severe clinical manifestations such as epilepsy.^12^, ^13^ Intriguingly, no spontaneous seizures have been detected in *Tsc2* ^*+/−*^ mice. It is known that dysregulated mTOR signaling disrupts normal cellular processes that not only contribute to the formation of cortical tubers^14^ but also to alterations in proliferation and synaptic plasticity, which influence neuronal excitability.^15^, ^16^, ^17^ Importantly, our group recently reported that female *Tsc2* ^*+/−*^ mice exhibit increased glutamate concentration in the prefrontal cortex (PFC).^18^ Furthermore, from a mechanistic standpoint, these models are also expected to have the propensity to show higher seizure susceptibility irrespective of the presence of lesions; however, little evidence is yet available to support this assumption. Given the high prevalence of *Tsc2* mutations and their association with severe epileptic episodes,^19^, ^20^ it is fundamental to characterize seizure susceptibility in the *Tsc2* ^*+/−*^ mouse model.

Concerning sexual dimorphism, clinical and preclinical studies suggest that gonadal hormone status influences seizure occurrence in women/females with epilepsy.^21^, ^22^, ^23^ Whereas estrogen has been identified as a proconvulsant, progesterone has been associated with anticonvulsant properties.^21^, ^22^, ^24^ Accordingly, hormonal fluctuations during the menstrual cycle have been shown to affect the frequency and severity of epileptic episodes.^21^ Furthermore, cumulative evidence suggests that behavioral outcomes differ across the circadian cycle in general epilepsy models.^25^, ^26^, ^27^ Therefore, for the one‐step characterization of *Tsc2* ^*+/−*^ mouse seizure vulnerability, it is fundamental to consider biological sex and the circadian cycle as preponderant variables.

To test the hypothesis of higher seizure susceptibility in the *Tsc2* ^*+/−*^ mouse model, acute models can be used as an initial screening tool before using long‐term models. And so, we investigated seizure susceptibility and severity in male and female *Tsc2* ^*+/−*^ mice induced by Kainic acid (KA), a potent neuroexcitatory amino acid, that is commonly used to induce seizures in animal models.^25^, ^28^ Latency times, seizure severity and frequency, and exploratory and anxiety‐like behavior were analyzed during two circadian periods (light and dark phases) before and after acute KA injection.

## 2 METHODS

### 2.1 Animals

Male and female heterozygous *Tsc2* ^*+/−*^ mice aged 8–12 weeks were used (*n* = 12 in each of 6 independent groups). *Tsc2* ^*+/−*^ mice were generated as previously described (genetic background: C57BL/6NCrl).^29^ The *Tsc2* ^*+/+*^ littermates were used as controls and referred as wild‐type (WT). Mice were housed at the Institute of Nuclear Science Applied to Health (ICNAS) animal facility at 22°C under a 12‐h light–dark cycle and provided ad libitum access to food and water. Experimental procedures were reviewed and approved by the Animal Welfare and Ethics Body of ICNAS following the guidelines of the European Community for the use of animals in the laboratory (86/609/EE) and the Portuguese law for the care and use of experimental animals (DL n° 129/92).

### 2.2 Kainic acid (KA) administration

Kainic acid (KA) (Biogen Cientifica S.L., Madrid, Spain) was dissolved in 0.9% sodium chloride (NaCl) solution (pH 7.3). Animals were administrated with an acute intraperitoneal injection (i.p.) of saline in a total volume of 100 μL per 25 g of animal weight, followed by a second injection of KA 20 mg/kg (i.p.) of the same volume.

### 2.3 Evaluation of seizure susceptibility

**FIGURE 1:**
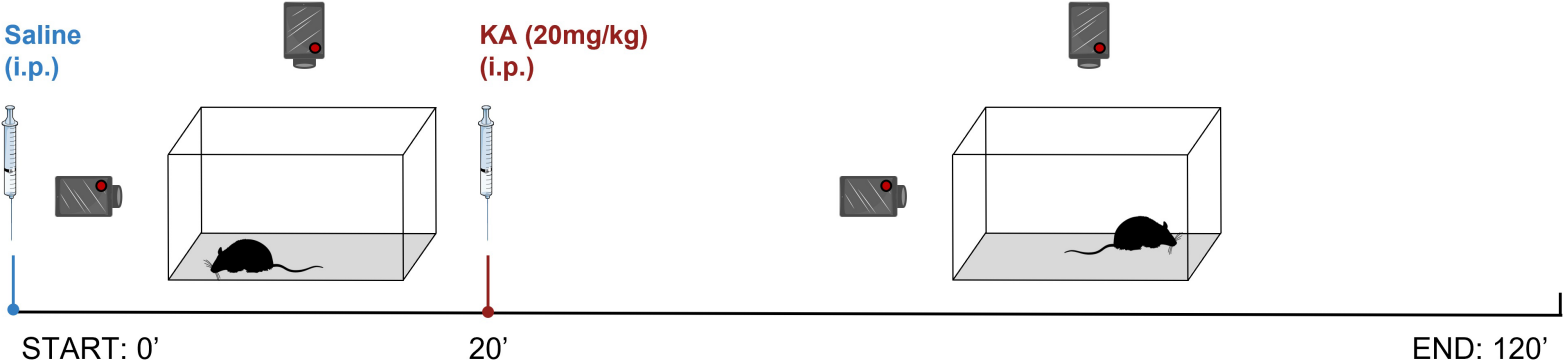
Schematic representation of experimental protocol used to characterize seizure susceptibility and severity to kainic acid. Male and female mice aged between 8 and 12 weeks were individually placed in an open‐field box (20 × 40 cm), video‐recorded (from top and front), and evaluated over 120 min: 20 min at control conditions following saline intraperitoneal (i.p.) injection and 100 min after a single kainic acid (KA) injection (20 mg/kg, i.p.). The figure was partly generated using Servier Medical Art, provided by Servier, licensed under a Creative Commons Attribution 3.0 unported license.

To study the effect of the circadian cycle on seizure susceptibility, experiments were performed during both light and dark phase, which corresponded to zeitgeber time (ZT) 6–8 and 14–16, respectively (ZT0: “lights on” onset). Each mouse was individually placed in an open‐field box (20 × 40 cm), video‐recorded (from top to front), and evaluated over 120 min: 20 min under control conditions following saline injection (i.p.) and 100 min after a single KA injection (20 mg/kg, i.p.) (Figure 1). Seizure severity was identified according to a modified Racine scale as previously described^30^ (Table S1) and a time frame of 20 min, 60 min post‐KA administration [time interval 80′ to 100′], was considered as the stabilization period of KA effect. Latency to the first seizure was considered for seizure susceptibility evaluation. Latency to the first seizure was considered for seizure susceptibility evaluation and defined as the first‐time animals registered a seizure‐related behavioral alteration (Table S1) characterized by a quick, sharp muscle movement, which looked like a twitch or a spasm face (i.e., stage 0.5–1.5).

### 2.4 Analysis of exploratory and anxiety‐like behavior

Mouse activity was monitored, and related metrics were extracted from top‐video recordings using ezTrack software.^31^ Videos were analyzed for 4 min at control and post‐KA conditions, 1 h after administration, to evaluate exploratory and anxiety‐like behavior. The metrics under study included total distance traveled (exploratory behavior) and the percentage of time animals spent at the center of the open field (anxiety‐related behavior).

### 2.5 Estrous cycle determination

A vaginal smear was collected, and cells were transferred to a dry glass slide and dried overnight.^32^ The glass slides were stained with crystal violet and examined by light microscopy (Carl Zeiss, Oberkochen, Germany). The estrous cycle stages were determined based on the presence or absence of specific cells according to^32^ (Figure S1). Next, we grouped the four stages of the estrous cycle into two main groups: the ovulatory phase (late diestrus, proestrus and estrus; progesterone decline and estrogen rising) and the post‐ovulatory phase (metestrus and diestrus; estrogen decline and progesterone rising).^33^ After estrous cycle segregation, groups were composed of females in ovulatory (Light phase: *n* = 7 WT, *n* = 6 *Tsc2* ^*+/−*^; Dark phase: *n* = 8 WT, *n* = 5 *Tsc2* ^*+/−*^) and post‐ovulatory phase (Light phase: *n* = 5 WT, *n* = 6 *Tsc2* ^*+/−*^; Dark phase: *n* = 4 WT, *n* = 7 *Tsc2* ^*+/−*^).

### 2.6 Statistical analysis

Statistical analyses were performed using GraphPad Prism version 8.0.1 (GraphPad Software, San Diego, California). All datasets were tested for normality with the Shapiro–Wilk test. Whenever normality criteria were met, analyses of variance were used. For non‐parametric data, quantile–quantile (Q‐Q) plots were evaluated, and parametric tests were used as observations lay approximately on the straight line of the Q‐Q plot. To explore the influence of genotype and sex over time, repeated measures 2‐WAY ANOVA with the Tukey test for multiple comparison correction was performed in the two circadian periods separately. Moreover, 3WAY ANOVA and ordinary 1WAY ANOVA with Sidak's test for multiple comparison correction were used to investigate the effect of the 3 factors (genotype, sex and circadian cycle) and the impact of KA, respectively. Outliers were identified using the ROUT method (Q = 5%), specifically 2 male WT, 1 male *Tsc2* ^*+/−*^, 1 female WT, and 2 female *Tsc2* ^*+/−*^.

## 3 RESULTS

### 3.1 Acute KA administration significantly increases seizure susceptibility in the *Tsc2* ^*+/−*^ mouse model in a sex‐ and circadian cycle‐dependent manner

**FIGURE 2:**
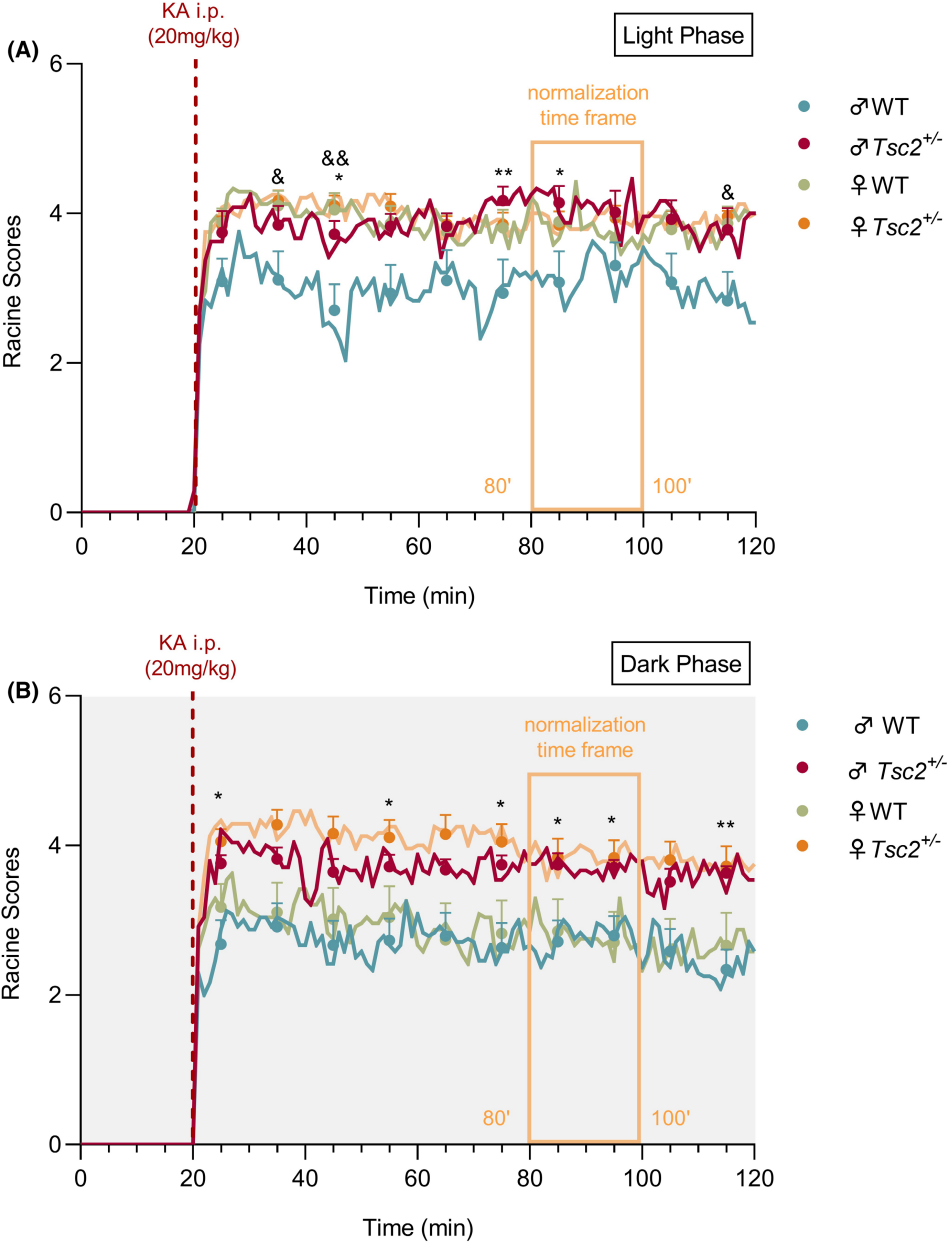
Acute KA administration significantly increases vulnerability in the TSC2 mouse model. The Racine scores were evaluated during 120 min of the experiment, following acute injection of saline and KA solution at (A) light and (B) dark phases. The red dashed line represents the time of KA injection, the light orange rectangular frame the time chosen to study seizure severity during stabilization of seizure occurrence (80–100 min), and the symbols are the mean values every 10 min between 20′ and 120′ (10 time points). For saline conditions, all mice exhibited normal behavior (Racine scores = 0) that changed post‐KA to a pattern of seizure events highlighted by susceptibility of *Tsc2* ^*+/−*^ animals for both light and dark phase. Post hoc tests following ANOVA revealed a sex‐dependent vulnerability for both light and dark periods as only *Tsc2* ^*+/−*^ males had significantly higher scores than WT males. Moreover, during the light phase in control animals, females exhibited significantly increased severity by a constant pattern of higher Racine scores compared with WT males following KA injection. The results are expressed as mean ± SEM (*n* = 12 for each group). *p < 0.05, **p < 0.01 comparing WT and *Tsc2* ^*+/−*^ males and ^&^ *p* < 0.05, ^&&^ *p* < 0.01 comparing WT males and WT females by 2WAY repeated measures ANOVA with Tukey's multiple comparisons‐test.

To evaluate susceptibility to KA‐induced seizures, *Tsc2* ^*+/−*^ and WT mice were injected with an acute administration of saline and KA. The mortality rate after KA administration was less than 0.1% and equal for both genotypes and sexes (0.083%). As expected, at saline conditions, all mice exhibited normal behavior (Racine scores = 0) that changed post‐KA injection to a pattern of seizure occurrence. After KA administration and for the two circadian periods separately, we found a significant difference among the groups being compared for both light (2WAY repeated measures ANOVA, group effect: *p* < 0.001, Figure 2A) and dark (2WAY repeated measures ANOVA, group effect: *p* = 0.0027, Figure 2B) phases. Further, the post hoc tests exposed an influence of genotype that was distinct between the two sexes. Indeed, *Tsc2* ^*+/−*^ animals showed an increase in KA susceptibility but, only *Tsc2* ^*+/−*^ males had consistently higher scores than their WT littermates at both light and dark phases (Figure 2). Moreover, during the light phase in control animals, females exhibited significantly increased severity by a constant pattern of higher Racine scores compared with WT males following KA injection (Figure 2A).

**FIGURE 3:**
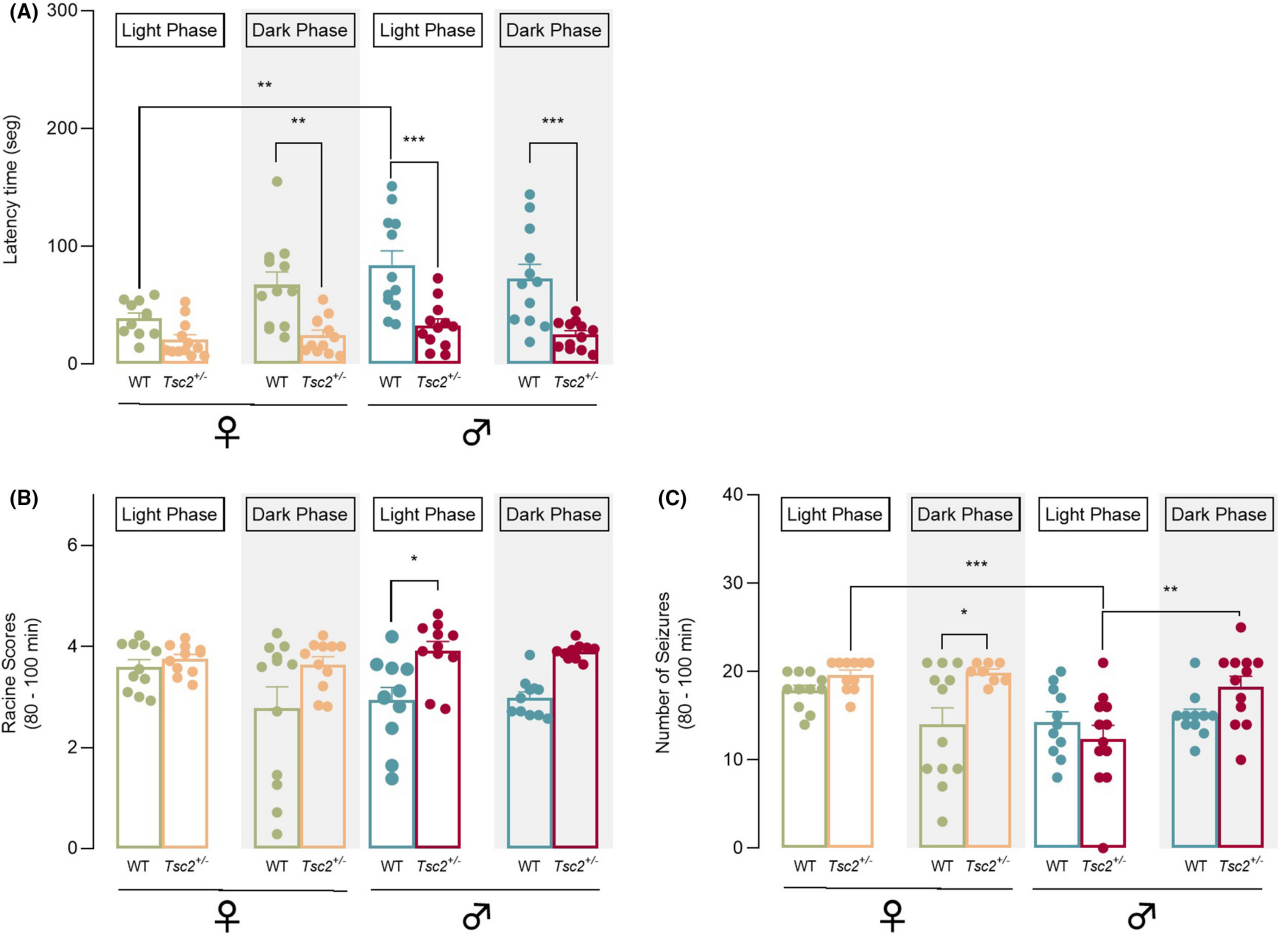
KA administration increases seizure vulnerability for *Tsc2* ^*+/−*^ mice in a sex and circadian cycle‐dependent matter. (A) Latency to the first seizure accordingly to each circadian cycle. Overall mutant mice had shorter latency times than their WT littermates that occurred in males for both circadian phases and in females during the dark phase. In control animals, males exhibited significantly higher latency times than females for the light phase. (B) Racine score values accordingly to each circadian cycle. Males during light phase showed a statistically significant difference between experimental groups, exposing an increased susceptibility for *Tsc2* ^*+/−*^ males compared with their WT littermates. (C) Number of seizures accordingly to each circadian cycle. *Tsc2* ^*+/−*^ females had higher number of seizures compared with their WT littermates during dark phase and compared with *Tsc2* ^*+/−*^ male during light phase. Moreover, for *Tsc2* ^*+/−*^ males, number of seizures was significantly increased during dark phase compared with light phase. The results are expressed as mean ± SEM (*n* = 8–12 for each group). *p < 0.05, **p < 0.01, ***p < 0.001 by 3‐WAY ANOVA with Sidak's multiple comparisons test correction.

Next, we focused on the onset and stabilization period (80′‐100′ min post‐KA injection) (Table S2). Overall, our data indicated that *Tsc2* ^*+/−*^ present higher susceptibility to KA‐induced seizures than their WT littermates, which differ in a sex‐ and circadian cycle‐dependent manner. It was observed that both genotype and sex significantly influenced the onset of the first seizure (3‐WAY ANOVA, genotype – *F*(1, 85) = 53.43, *p* < 0.001; sex – *F*(1, 85) = 9.208, *p* = 0.0032, Figure 3A). Moreover, a significant interaction between sex and circadian cycle has been noted (3‐WAY ANOVA, sex × circadian cycle – *F*(1, 85) = 6.287, *p* = 0.0141, Figure 3A). The post hoc tests further revealed that, while *Tsc2* ^*+/−*^ males exhibited shorter latency times at both light and dark phases compared with WT littermates, *Tsc2* ^*+/−*^ females only present shorter latencies at dark phase (Figure 3A). In agreement with this observation, during the light phase, WT females exhibited significantly reduced latency times than WT males which resemble what had been previously observed (Figure 2A). In Figure 3B, seizure severity was represented by Racine score, and we found a significant effect of genotype (3‐WAY ANOVA, genotype – *F*(1, 80) = 22.65, *p* < 0.0001, Figure 3B). Nevertheless, post hoc tests showed that only males during light phase had a statistically significant difference between experimental groups, exposing an increased susceptibility for *Tsc2* ^*+/−*^ males compared with their WT littermates (Figure 3B).

For seizure frequency during the stabilization period, it was observed that both genotype and sex had a significant effect (3‐WAY ANOVA, genotype – *F*(1, 80) = 6.913, *p* = 0.0103; sex – *F*(1, 80) = 11.23, *p* = 0.0012, Figure 3C). Moreover, a significant interaction between genotype and circadian cycle and sex and circadian cycle has been noted (3‐WAY ANOVA, genotype × circadian cycle – *F*(1, 80) = 7.29, *p* = 0.0085; sex × circadian cycle – *F*(1, 80) = 8.913, *p* = 0.0038, Figure 3C). The post hoc tests further revealed that *Tsc2* ^*+/−*^ females had more seizures compared with their WT littermates during dark phase and with *Tsc2* ^*+/−*^ males during light phase. Moreover, for *Tsc2* ^*+/−*^ males, seizures significantly increased during dark phase (Figure 3C).

### 3.2 Anxiety levels post‐KA are influenced by sex and circadian cycle in a genotype‐dependent manner

**FIGURE 4:**
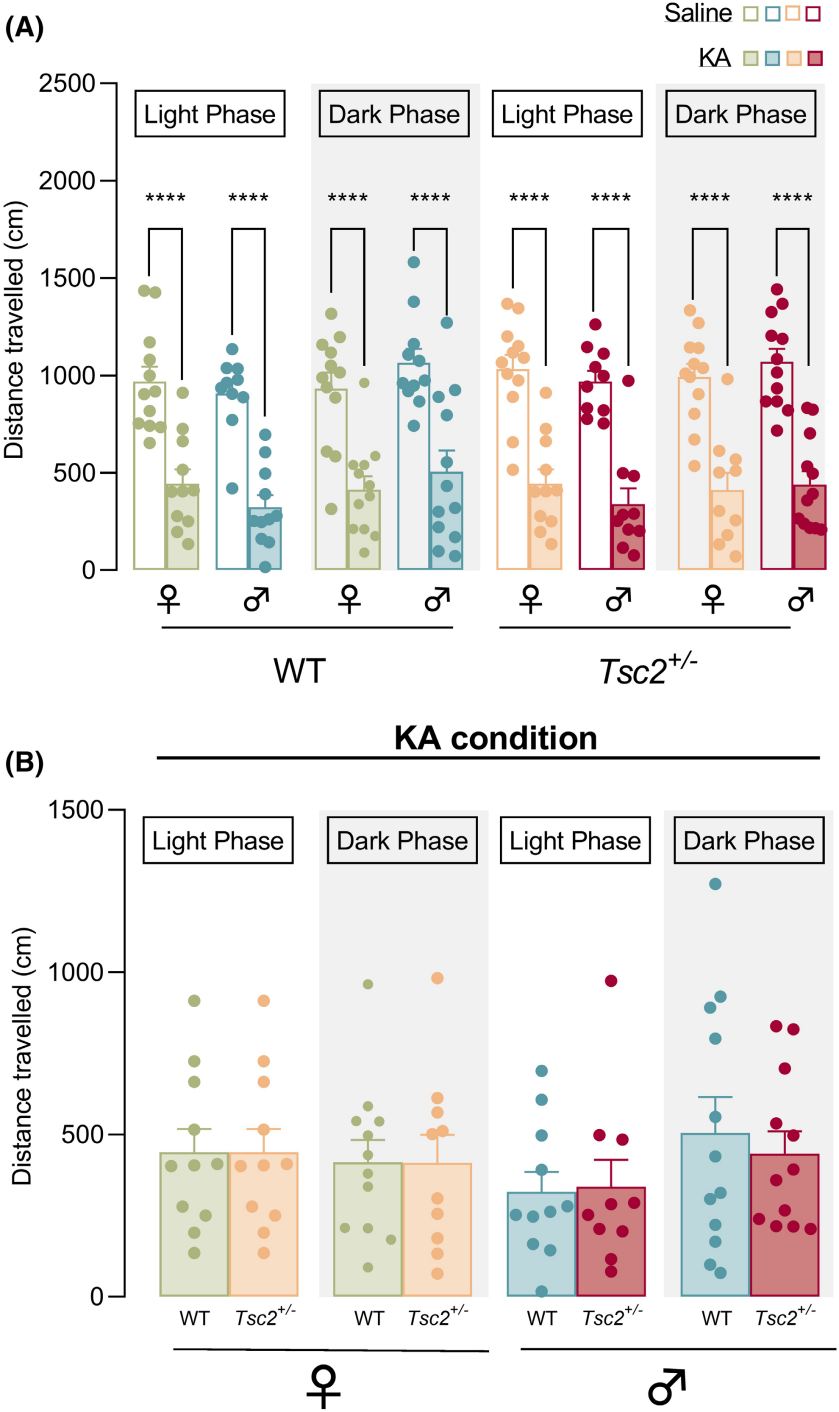
Exploratory behavior decreases significantly and similarly post‐KA injection across all groups. (A) Total distance traveled for each circadian cycle and condition (saline and KA). All animals at saline conditions had similar distances traveled in open field arena that decreased significantly post‐KA injection at both light and dark phase. (B) Total distance traveled for each circadian cycle for KA condition. No significant differences were reported between experimental groups post‐KA injection. The results are expressed as mean ± SEM (*n* = 10–12 for each group). *p < 0.05, **p < 0.01, ***p < 0.001, ****p < 0.0001 by Ordinary 1‐WAY ANOVA (A) and 3‐WAY ANOVA (B) with Sidak's multiple comparisons test.

**FIGURE 5:**
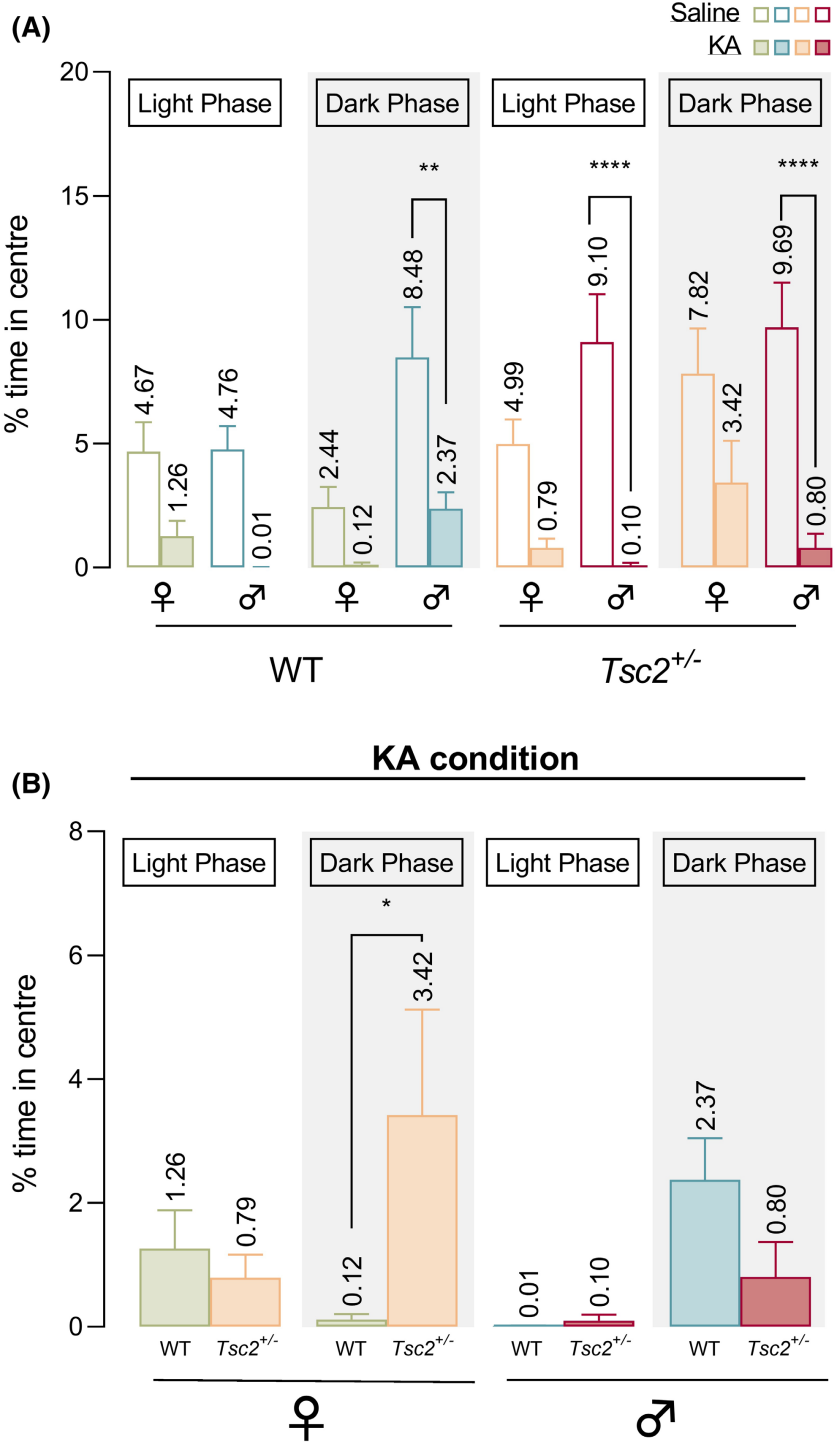
Anxiety levels post‐KA are influenced by sex and circadian cycle in a genotype‐dependent manner. (A) Percentage of time in the center of the open field for each circadian cycle and condition (saline and KA). *Tsc2* ^*+/−*^ animals showed a significant increase of anxiety‐like behavior, comparing saline and post‐KA condition, that generally was not observed for WT mice. (B) Percentage of time in the center of the open field for each circadian cycle for KA condition. WT females exhibited lower anxiety levels during dark phase compared with *Tsc2* ^*+/−*^ females. The results are expressed as mean ± SEM (*n* = 8–12 for each group). *p < 0.05, **p < 0.01, ***p < 0.001, ****p < 0.0001 by Ordinary 1‐WAY ANOVA (A) and 3‐WAY ANOVA (B) with Sidak's multiple comparisons test.

To explore the impact that seizure occurrence has on neurobehavioral comorbidities, we further evaluated locomotion and anxiety‐like behavior (Figures 4 and 5; Tables S3 and S4). Firstly, we compared saline and KA conditions for the total distance traveled and found a significant group effect (Ordinary 1WAY ANOVA, *p* < 0.0001, Figure 4A). Moreover, post hoc tests revealed that, all animals at saline conditions had similar distances traveled that decreased significantly post‐KA injection at both light and dark phase (Ordinary 1WAY ANOVA, *p* < 0.0001 for all saline versus KA comparisons, Figure 4A). After KA administration, we reported no significant effect of genotype, sex nor circadian cycle for total distance traveled (3‐WAY ANOVA; Figure 4B).

Additionally, anxiety‐like behavior was evaluated by comparing the percentage of time each animal stayed in the center of the open field (Figure 5; Table S4). Once again, a group effect has been found when directly comparing saline and KA conditions (Ordinary 1WAY ANOVA, *p* < 0.0001, Figure 5A). Predictably, all experimental groups spent more time in center of the field for saline conditions than under KA effect. Interestingly, comparing saline and KA condition, post hoc tests revealed that *Tsc2* ^*+/−*^ animals showed a significant increase of anxiety‐like behavior (spent less time in the center) that generally was not observed for WT mice (Figure 5A). Furthermore, after KA administration, we found that anxiety levels post‐KA were influenced by circadian cycle (3‐WAY ANOVA, *F*[1, 65] = 4.090, *p* = 0.0473, Figure 5B). Moreover, a significant interaction between genotype, sex, and circadian cycle (3‐WAY ANOVAF[1, 65] = 5.842, *p* = 0.0185, Figure 5B) has been noted. In fact, post hoc tests for post‐KA condition, revealed that during dark phase, WT females exhibited significantly lower anxiety levels than *Tsc2* ^*+/−*^ females (Figure 5B).

### 3.3 Estrous cycle influenced severity to KA‐induced seizures in a genotype‐dependent manner

**FIGURE 6:**
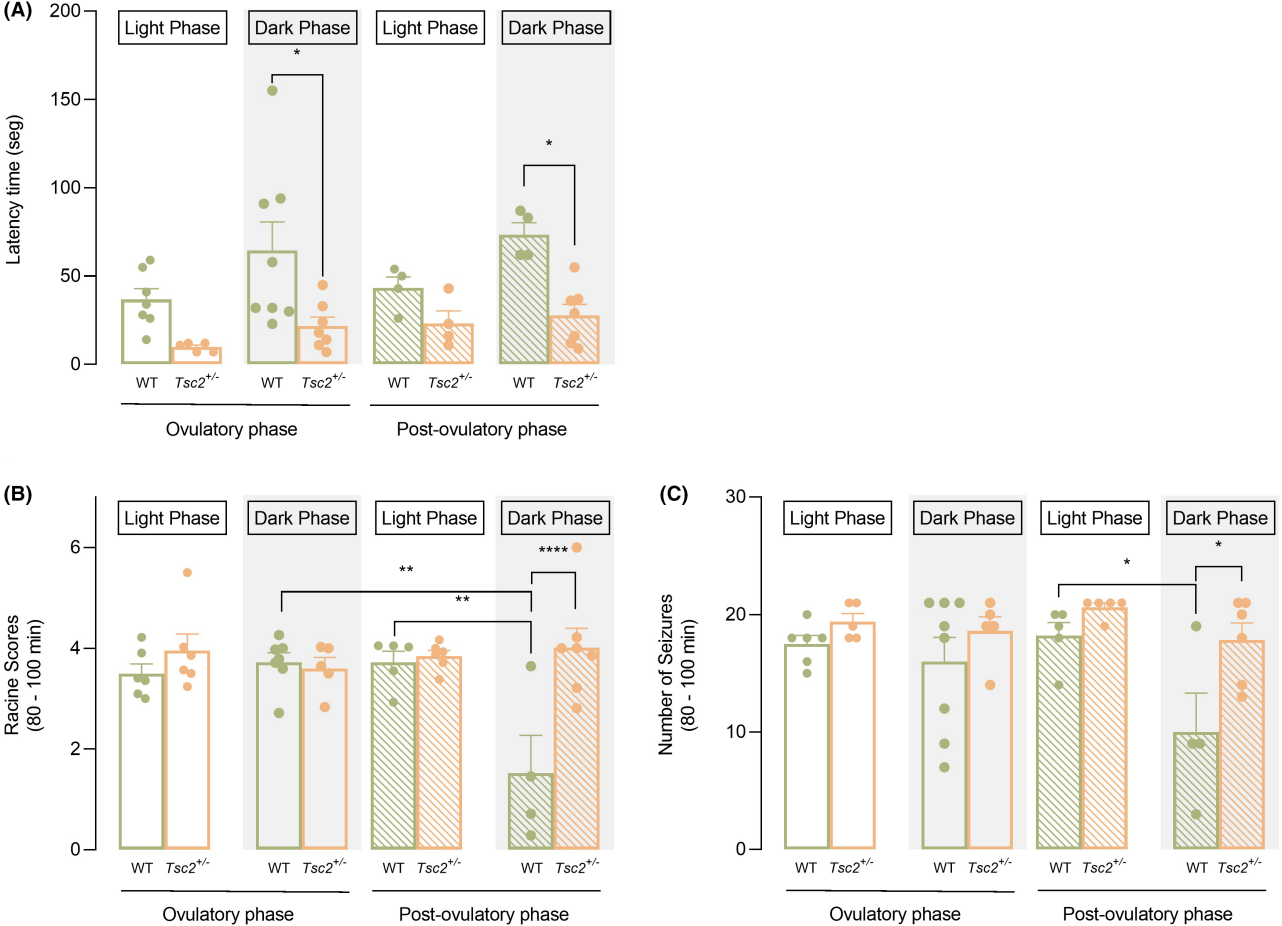
Estrous cycle influenced females' severity to KA in a genotype‐dependent manner. (A) Latency to the first seizure accordingly to each circadian cycle. Latency to the first seizure was significantly reduced for *Tsc2* ^*+/−*^ females compared with WT animals at the both ovulatory and post‐ovulatory phases during the dark phase. (B) Racine score values accordingly to each circadian cycle. Racine scores were significantly increased in the mutant female compared with WT at the post‐ovulatory stage during the dark phase. Moreover, in control animals, Racine scores were significantly higher in the ovulatory phase compared with post‐ovulatory phase during dark phase and during light phase compared with dark phase for post‐ovulatory phase. (C) Number of seizures accordingly to each circadian cycle. *Tsc2* ^*+/−*^ females in post‐ovulatory stage had higher number of seizures compared with their WT group during dark phase. Moreover, for WT animals in post‐ovulatory phase, number of seizures was significantly increased during light phase compared with dark phase. The results are expressed as mean ± SEM (*n* = 4–8 for each group). *p < 0.05, ***p < 0.001, ****p < 0.0001 by 3‐WAY ANOVA with Sidak's multiple comparisons test.

Finally, to discriminate the influence of the estrous cycle, we segregated females on ovulatory and post‐ovulatory phases (Figure 6; Table S5).

It was detected that latency time was influenced by genotype and circadian cycle (3‐WAY ANOVA, genotype – *F*(1, 38) = 22.35, *p* < 0.001; circadian cycle – *F*(1, 38) = 6.731, *p* = 0.0134, Figure 6A), while no effect of estrous cycle phase was noted. Specifically, differences were observed between experimental groups during the dark phase. In fact, WT females display a later onset of seizures compared with *Tsc2* ^*+/−*^ females in both ovulatory and post‐ovulatory phases for that circadian phase (Figure 6A). Regarding the severity of seizure, Racine scores showed an effect of genotype and circadian cycle (3‐WAY ANOVA, genotype – *F*(1, 37) = 11.37, *p* = 0.0018; circadian cycle – *F*(1, 37) = 6.186, *p* = 0.0175, Figure 6B). Interestingly, in this parameter, significant interactions between all factors under study (3‐WAY ANOVA, genotype × estrous cycle – *F*(1, 37) = 5.936, *p* = 0.0198; genotype × circadian cycle – *F*(1, 37) = 4.358, *p* = 0.0438; genotype × circadian cycle × estrous cycle phase – *F*(1, 37) = 9.99, *p* = 0.0031, Figure 6B) were found. Moreover, WT female mice in post‐ovulatory and at dark phase exhibited less severe seizure occurrence compared with *Tsc2* ^*+/−*^ females (Figure 6B). Further, the post‐ovulatory phase appears to be protective to WT females especially at dark phase. Explicitly, WT females in the post‐ovulatory phase and for dark phase showed less severe episodes of seizures compared with the ovulatory phase (Figure 6B). Moreover, WT females in the post‐ovulatory phase were less susceptible during dark phase compared with light phase (Figure 6B); the same was not reported for mutant animals.

Next, it was observed that both genotype and circadian cycle significantly influenced seizure frequency (3‐WAY ANOVA, genotype – *F*(1, 35) = 10.22, *p* = 0.0029; circadian cycle – *F*(1, 35) = 8.321, *p* = 0.0067, Figure 6C) but no significant effect of estrous cycle was found for that measure. The post hoc tests further revealed that *Tsc2* ^*+/−*^ females at post‐ovulatory stage had a higher number of seizures compared with their WT littermates during dark phase. Moreover, in post‐ovulatory phase and for WT females, the number of seizures was significantly increased during light phase compared with dark phase (Figure 6C).

Ultimately, we also explored the possibility of estrous cycle influence on exploratory and anxiety‐like behavior (Figures S2 and S3; Tables S7 and S8). Once again, comparing both conditions (saline vs. KA), after estrous cycle segregation we found a significant group effect for the total distance traveled (Ordinary 1WAY ANOVA, *p* < 0.0001, Figure S2a). Moreover, the post hoc tests revealed that, both the estrous cycle phases displayed a similar reduction in the total distance traveled in the open field for the light phase that was significant for WT and *Tsc2* ^*+/−*^ animals in the post‐ovulatory stage (Figure S2a). Moreover, during dark phase, WT animals reduced the distance they traveled during the ovulatory stage, while *Tsc2* ^*+/−*^ mice reduced in both estrous cycle phases. After KA administration, there was no significant effect on any of the three factors under study (3‐WAY ANOVA, Figure S2b).

For the percentage of time in the center of the open field, once again, we found a significant effect of group comparing saline and KA conditions (Ordinary 1WAY ANOVA, *p* = 0.0023; Figure S3a). Furthermore, the post hoc tests revealed that the percentage of time in the center was only significantly reduced (saline vs. KA) for *Tsc2* ^*+/−*^ female mice during the dark phase at the post‐ovulatory phase (Figure S3a). After KA administration, once again, there was no significant effect of any of the three factors under study (3‐WAY ANOVA, Figure S3b).

## 4 DISCUSSION

Our work provides a detailed account of seizure susceptibility in response to a proconvulsant drug in the TSC2 mouse model, focusing on the influence of biological sex and circadian cycle. Our results demonstrate that *Tsc2* ^*+/−*^ mice, post‐KA injection, require less time to present the first seizure and display more severe and frequent seizures compared with control animals. Based on evidence that the TSC2 animal model did not show significant pathological brain abnormalities,^8^ our data support the notion that severity of seizures in TSC cannot be exclusively attributed to focal lesions in brain tissue, such as cortical tubers, which are commonly associated with epileptic onset.^2^, ^4^, ^6^, ^7^ In other words, direct physiological impairments at cellular and circuit levels contribute irrespective of the presence of overt lesions. Furthermore, according to our previous data that showed that *Tsc2* ^*+/−*^ female mice exhibit increased cortical glutamate concentration,^18^ our results on acute KA administration, a potent neuroexcitatory amino acid, suggest that disruption of *Tsc2* allele exacerbates the mechanisms of seizure occurrence.

Remarkably, apart from the genetic background, we consistently found a significant effect of sex and the circadian cycle on vulnerability to KA‐induced seizures. Accordingly, some studies have reported that several behavioral and physiological differences between sexes are dependent on the circadian cycle.^34^, ^35^ However, although some previous studies have explored the involvement of these factors in induced epileptic models separately,^26^, ^27^, ^36^, ^37^, ^38^, ^39^ none have so far considered both factors in the same study.

It has been showed that the circadian phases can impact neuronal excitability by causing oscillations in the E/I balance with synaptic excitation being exacerbated during the light phase.^27^ Accordingly, several studies using epileptic agents have demonstrated that animals are more vulnerable to seizures during the light phase.^26^, ^36^ On other side, numerous publications support sex differences in seizure susceptibility.^40^ Indeed, most data agree that, in experimental models, females are more vulnerable than males.^37^, ^38^, ^39^ Here we explore the involvement of those factors (sex and circadian cycle) together and found that generally females were more vulnerable to KA induced seizures than males from both genotypes during the light phase, which agrees with the above‐mentioned studies on circadian cycle and biological sex. Moreover, while *Tsc2* ^*+/−*^ males, compared with their controls, showed earlier seizure onset and aggravated seizure progression for both circadian phases, *Tsc2* ^*+/−*^ females only presented increased susceptibility for seizure onset and frequency during the dark phase. From those results, it is possible to infer that sexual dimorphisms exclusively occurred during light phase independently of animal's genetic background. Interestingly, while genotype‐specific differences in males were mostly not affected by circadian cycle, those differences in females were only present during dark phase, the circadian phase that corresponds to the absence of sexual dimorphisms.

In females it has been proven that hormonal fluctuations can regulate circadian responses^41^ and also interfere with epileptic episodes.^21^, ^22^, ^42^ In fact, it is widely recognized that estrogen has proconvulsant while progesterone has anticonvulsant properties.^22^ In specific that estrogen rising and progesterone decline aggravate seizure exacerbations and frequency in epileptic patients.^21^, ^24^ Here, we found no significant effect of estrous cycle on seizure onset or frequency. Still, we reported a significant interaction between genotype, circadian phase, and estrous cycle stage for seizure severity. Specifically, vulnerability in WT female mice was intensified during the ovulatory phase, corresponding to progesterone decline and estrogen rise. Yet, that difference was only reported during the dark phase. Moreover, we reported increased severity during light phase compared with dark phase during post‐ovulatory stage. These results indicate that the post‐ovulatory phase provides protection for control females. Nevertheless, the circadian rhythm appears to have a stronger impact in here, affecting this protection in WT females during light phase.

In contrast, even though for WT animals, females appear to be less vulnerable during the post‐ovulatory stage, the same was not observed in *Tsc2* ^*+/−*^ mice. A possible explanation for this distinct effect may be due to the fact that progesterone has been proven to be a suppressor of mTOR levels.^43^, ^44^, ^45^, ^46^ As for *Tsc2* ^*+/−*^ animals, there is a hyperactivation of mTOR pathway, the anticonvulsant effects of progesterone may be abolished as there is a parallel mechanism involving this hormone action. Ultimately, we can say that these results suggest that female vulnerability could be a result of hormonal fluctuations as well as the circadian cycle changes that highly depends on their genetic background. Moreover, our findings emphasize the importance of considering the estrous cycle phase and the circadian cycle in future investigations of epilepsy in this animal model. To our knowledge, this is the first data on how the estrous and circadian cycle affects seizure vulnerability in the TSC2 mouse model for KA‐induced seizures. Nevertheless, we acknowledge the preliminarily of our conclusions based on a small sample size for estrous cycle. We reinforce that further studies should be set to better address female's cycle influence.

From exploratory behavior analysis, *Tsc2* ^*+/−*^ animals exhibited a significant increase in anxiety‐like behavior post‐KA compared with their saline condition, which generally was not observed for WT mice. In line with this result, a previous study reported increased anxiety‐related behaviors in a *Tsc2* mouse model.^47^ Furthermore, we found that anxiety levels post‐KA were significantly influenced by circadian cycle and by an interaction between genotype, sex, and circadian cycle. In fact, sexual dimorphism in behavior outcomes has been reported in the literature,^18^, ^48^, ^49^, ^50^ but that did not account for the influence of circadian cycle. Specifically, it has been reported that female C57BL/6J mice displayed increased anxiety in different behavioral tests compared with males.^48^, ^49^ Moreover, previous data also support sexual dimorphism in behavior outcomes for the TSC2 mouse model, characterized by elevated anxiety levels for mutant females described as a stress‐coping strategy.^18^, ^50^ Accordingly, here, we only found a significant increase in anxiety for *Tsc2* ^*+/−*^ females compared to their control group. Yet, that specific difference was only reported during dark phase. And so, it appears that circadian cycle strongly affects anxiety‐like behavior according to the genetic background and biological sex. Nevertheless, as we further exposed that sex significantly interacts with genotype and circadian cycle for anxiety‐like behavior, apart from the circadian cycle, once again, hormonal levels in females could be preponderant factors for anxiety‐like behavior and sexual dimorphisms for this measure. However, overall, we did not find a significant impact of the estrous cycle phase on behavior outcomes after KA administration. It is worth noting that the relationship between *Tsc2* mutation/mTOR pathway and estrogen/progesterone is still poorly understood, and further studies specifically focused on the role of this hormones are needed.

## 5 SIGNIFICANCE

Our work demonstrated for the first time the susceptibility of the TSC2 mouse model to KA‐induced seizures and disclosed the effects of sex and the circadian cycle. Our results showed that genetic background significantly affects seizure susceptibility to KA administration. Specifically, although the TSC2 mouse model did not have spontaneous epileptic events or significant pathological brain abnormalities, we showed that *Tsc2* ^*+/−*^ animals are more vulnerable to KA‐induced seizures, indicating that other neurological alterations may be involved in seizure occurrence. And so, our study suggested that induced models of epilepsy/seizures in *Tsc2* ^*+/−*^ mice can be used to study some aspects involving epilepsy for this disorder. Additionally, in the TSC2 mouse model, in response to the proconvulsant drug, we found evidence for the involvement of the circadian cycle and biological sex in seizure occurrence and anxiety‐like behavior.

In summary, our results validate that *Tsc2* ^*+/−*^ mice are more vulnerable to display seizures after an acute administration of a proconvulsant drug and support the use of the TSC2 mouse model in future TSC‐related induced epilepsy research. Moreover, here we highlight the importance of assessing male and female mice and circadian cycle periods separately. Future studies using this model should consider these variables to develop future therapeutic strategies for TSC‐related epilepsy.

## AUTHOR CONTRIBUTIONS

Mariana Lapo Pais performed experiments and analyzed data. Mariana Lapo Pais, Miguel Castelo‐Branco and Joana Gonçalves designed the project and prepared the manuscript. Joana Gonçalves supervised the project. Mariana Lapo Pais, João Martins, Miguel Castelo‐Branco and Joana Gonçalves discussed results and reviewed the manuscript. All authors read and approved the final manuscript.

## FUNDING INFORMATION

This work was supported by Ph.D. Fellow 2020.06582.BD (10.54499/2020.06582.BD) from FCT and Strategic Plan UIDP/04950/2020, COMPETE and FEDER funds, FCT, Portugal, ICNAS‐P.

## CONFLICT OF INTEREST STATEMENT

None of the authors have any conflict of interest. We confirm that we have read the Journal's position on issues involved in ethical publication and affirm that this report is consistent with those guidelines.

## Supporting information


Appendix S1

## Data Availability

All data needed to evaluate the conclusions in the paper are present in the paper and/or Appendix S1. Additional data related to this paper may be requested legally from the corresponding authors.
