# Supplementary material for: Increased susceptibility to kainate‐induced seizures in a mouse model of tuberous sclerosis complex: Importance of sex and circadian cycle
Source: Epilepsia Open. 2024 Jul 15;9(5):1710–22. doi: 10.1002/epi4.12955 (PMC11450656; doi:10.1002/epi4.12955)
Supplement: Supplementary file 1 — Appendix S1 [file EPI4-9-1710-s001.docx]

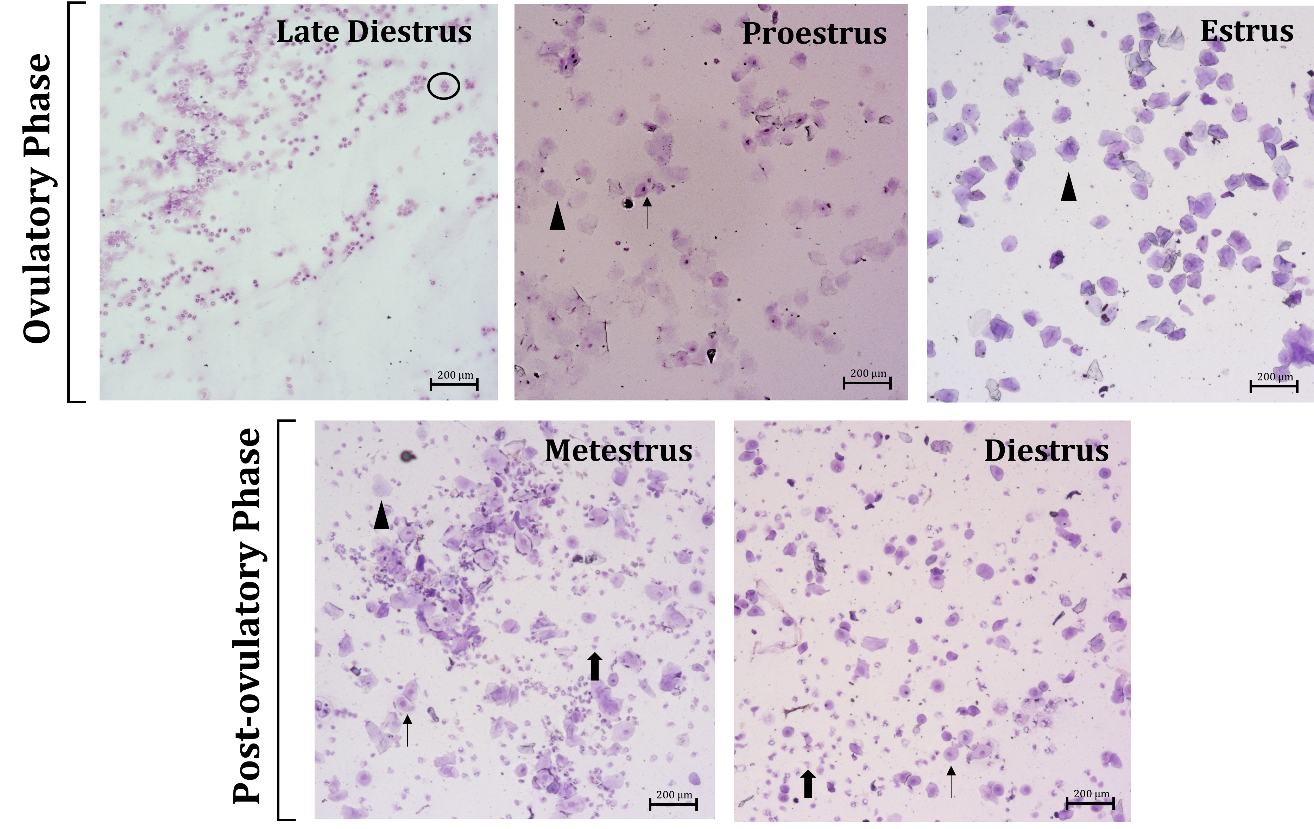


Figure S1. Vaginal cytological representation of female oestrous cycle. Three cell types are identified: leukocytes (), cornified epithelial () and nucleated epithelial (). When the female is in proestrus, mostly nucleated and some cornified epithelial cells are present. As the stage of the cycle advances to estrus, mostly cornified epithelial cells are present. Metestrus is a brief stage with cornified epithelial cells and leukocytes present. In diestrus, leukocytes are the predominant cell type, with few epithelial cells also present. Vaginal swabs during late diestrus show primarily polymorphonuclear leukocytes (○) (31). Scale bar = 200 µm.

**
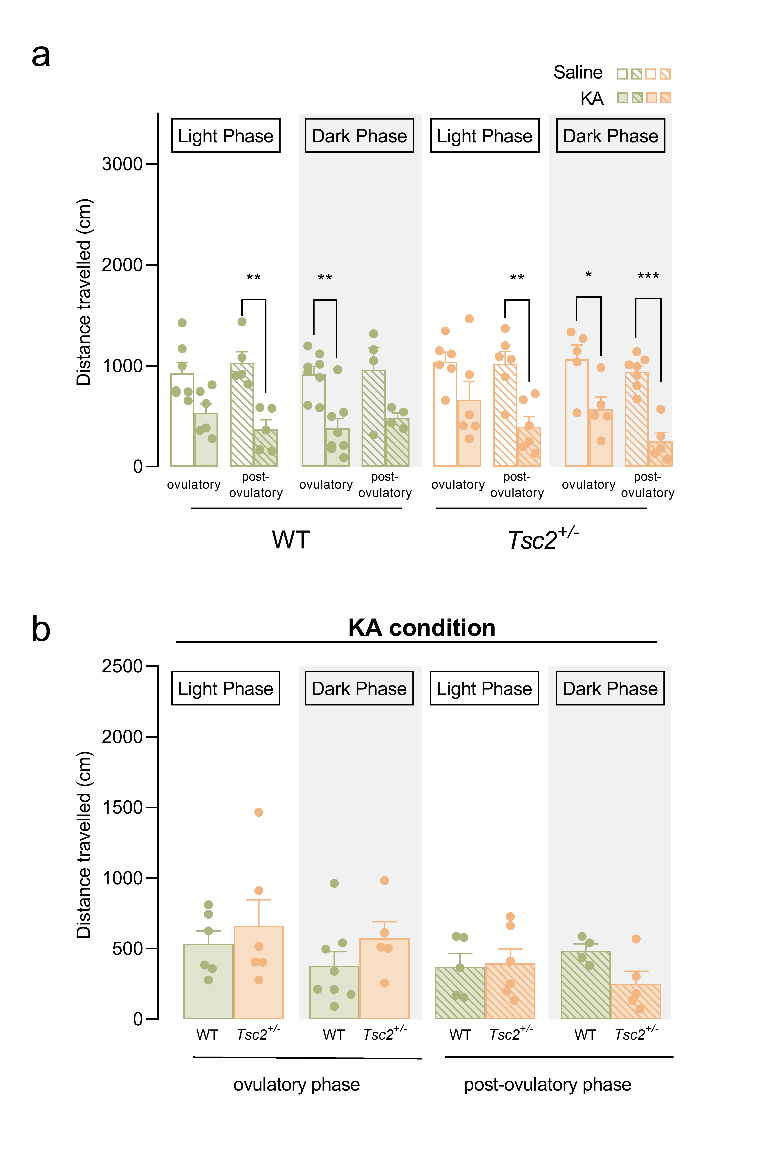
**

**Figure S2.** No impact of the oestrous cycle phase on exploratory behaviour. (a) Total distance travelled according to each oestrous cycle phase, circadian cycle and condition (saline and KA). Both the oestrous cycle phases displayed a similar reduction in the total distance travelled in the open field for the light phase that was significant for WT and *Tsc2^+/-^* animals in post-ovulatory stage. Moreover, during dark phase, WT animals reduced their distance travelled during ovulatory stage while *Tsc2^+/-^* mice reduced in both oestrous cycle phases. (b) Percentage of time in the centre of the open field according to each oestrous cycle phase and circadian cycle for KA condition. After KA administration, anxiety-like behaviour was similar for all oestrous cycle and circadian cycle phases. The results are expressed as mean ± SEM (n = 4 - 8 for each group). *p<0.05, **p<0.01, ***p<0.001, by Ordinary 1-WAY ANOVA (a) and 3-WAY ANOVA (b) with Sidak´s multiple comparisons test.


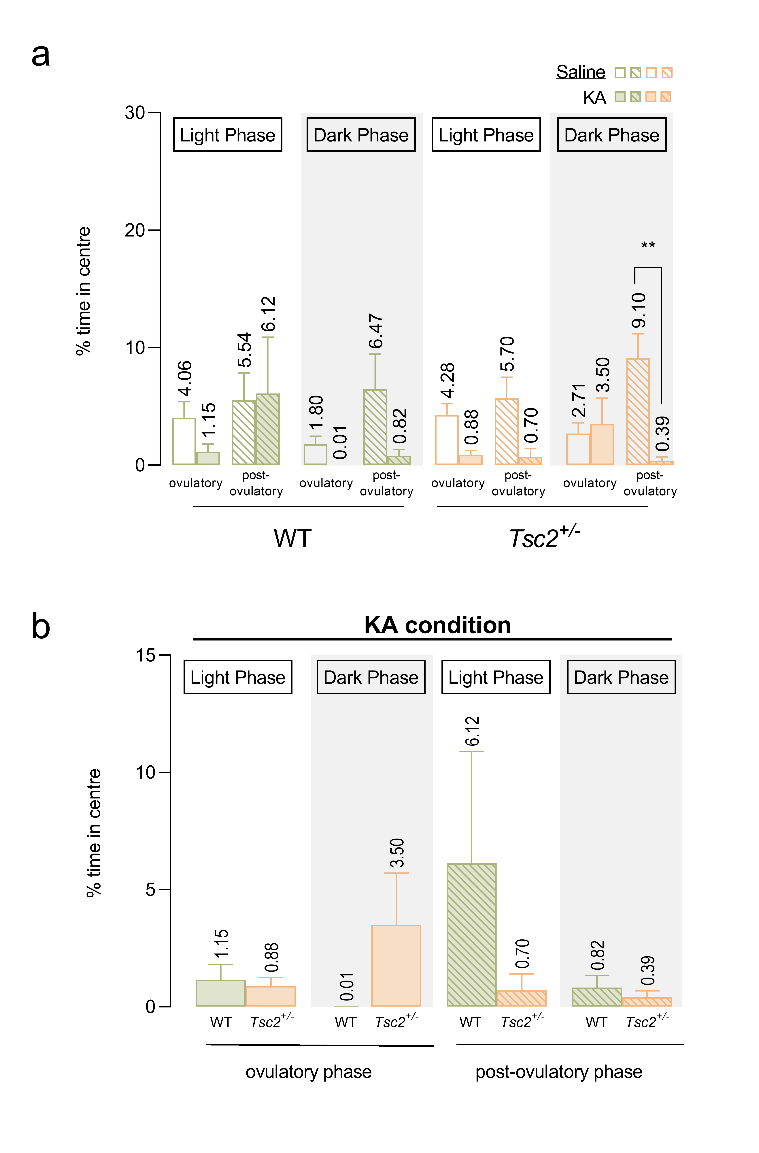


**Figure S3.** No impact of the oestrous cycle phase on anxiety-like behaviour. (a) Percentage of time in the centre of the open field according to each oestrous cycle phase, circadian cycle and condition (saline and KA). The percentage of time in the centre was only significantly reduced (saline vs KA) during the dark phase for *Tsc2^+/-^* mice at the post-ovulatory phase. (b) Percentage of time in the centre of the open field according to each oestrous cycle phase and circadian cycle for KA condition. After KA administration, anxiety-like behaviour was similar for all oestrous cycle and circadian cycle phases. The results are expressed as mean ± SEM (n = 4 - 7 for each group). *p<0.05, **p<0.01, ***p<0.001, by Ordinary 1-WAY ANOVA (a) and 3-WAY ANOVA (b) with Sidak´s multiple comparisons test.

**Table S1** Racine scoring system to evaluate KA-induced seizure severity of animals previously adapted from (29).

| **Score** | **Descriptions** | **Seizure correlation** |
| --- | --- | --- |
| 0 | No Response to KA. | No seizures |
| 0.5 | Short-term twitching of the ear and face. | Myoclonic seizure:  (Quick, sharp muscle movement, which often looks like a twitch or a spasm; They’re usually minor and brief) |
| 1 | Long-term twitching of the ear and face. |  |
| 1.5 | Twitching of the ear and face to the body. |  |
| 2 | Short-term myoclonic reflexes in the body. |  |
| 2.5 | Recurrent quick and severe myoclonic reflexes in the body. |  |
| 3 | Short-term rearing-up on hind-legs. |  |
| 3.5 | Severe rearing-up on hind-legs and transition to clonic seizure. | Clonic seizures  (Periods of shaking or jerking parts on the body; They’re usually long in their duration) |
| 4 | Short-term tonic-clonic seizures and normalization. |  |
| 4.5 | Long-term tonic-clonic seizures. |  |
| 5 | Severe recurrent generalized tonic-clonic seizures. |  |
| 5.5 | Prolonged generalized tonic-clonic seizures and unconsciousness. |  |
| 6 | Death. | No seizures |

KA: Kainic Acid.

**Table S2** Seizure susceptibility induced by KA injection. Mean values of latency times, Racine scores and number of seizures, 95% confidence intervals and respective P values obtained by 3WAY ANOVA with Sidak's multiple comparisons-test at light and dark phases for both genotypes according to each sex.

|  | **Circadian cycle** | **Genotype** | **Sex** | **N** | **Mean Value**  **± SEM** | **95 % CI** | **P** |
| --- | --- | --- | --- | --- | --- | --- | --- |
| **Latency Times (s)** | Light phase | WT | M | 12 | 84.3 ± 11.9 | 57.99 - 110.5 | **0.0002** |
|  |  | *Tsc2^+/-^* |  | 12 | 32.8 ± 5.7 | 20.41 - 45.26 |  |
|  |  | WT | F | 12 | 39.1 ± 4.4 | 29.28 – 48.90 | 0.4346 |
|  |  | *Tsc2^+/-^* |  | 12 | 14.9 ± 11.8 | 9.09 – 20.71 |  |
|  | Dark phase | WT | M | 12 | 72.9 ± 11.8 | 47 – 98.83 | **0.0006** |
|  |  | *Tsc2^+/-^* |  | 12 | 25.0 ± 3.4 | 17.43 – 32.57 |  |
|  |  | WT | F | 12 | 67.4 ± 10.9 | 43.50 – 91.34 | **0.0029** |
|  |  | *Tsc2^+/-^* |  | 12 | 24.5 ± 4.5 | 14.71 – 34.29 |  |
| **Racine Scores** | Light phase | WT | M | 11 | 2.9 ± 0.3 | 2.359 – 3.507 | **0.0229** |
|  |  | *Tsc2^+/-^* |  | 11 | 3.9 ± 0.2 | 3.507 – 4.320 |  |
|  |  | WT | F | 11 | 3.6 ± 0.1 | 3.281 – 3.914 | >0.99 |
|  |  | *Tsc2^+/-^* |  | 11 | 3.8 ± 0.1 | 3.553 – 3.949 |  |
|  | Dark phase | WT | M | 11 | 3.0 ± 0.1 | 2.729 – 3.237 | 0.0520 |
|  |  | *Tsc2^+/-^* |  | 10 | 3.9 ± 0.1 | 3.787 – 4.013 |  |
|  |  | WT | F | 12 | 2.8 ± 0.4 | 1.866 – 3.702 | 0.0599 |
|  |  | *Tsc2^+/-^* |  | 11 | 3.6 ± 0.1 | 3.312 – 3.978 |  |
| **Number of Seizures** | Light phase | WT | M | 11 | 14.27 ± 1.2 | 11.65 – 16.89 | 0.9668 |
|  |  | *Tsc2^+/-^* |  | 12 | 12.33 ± 1.6 | 8.892 – 15.77 |  |
|  |  | WT | F | 11 | 17.82 ± 0.6 | 16.45 – 19.19 | 0.983 |
|  |  | *Tsc2^+/-^* |  | 11 | 19.64 ± 0.5 | 18.46 – 20.81 |  |
|  | Dark phase | WT | M | 11 | 15.0 ± 0.8 | 13-33 – 16.67 | 0.4876 |
|  |  | *Tsc2^+/-^* |  | 12 | 18.25 ± 1.2 | 15.61 – 20.89 |  |
|  |  | WT | F | 12 | 14.0 ± 1.9 | 9.882 – 18.12 | **0.0213** |
|  |  | *Tsc2^+/-^* |  | 8 | 19.88 ± 0.4 | 18.93 – 20.82 |  |

CI: Confidence Interval; F: Female; KA: Kainic Acid; M: Male; N: Sample size; S: seconds; SEM: Standard Error of the Mean.

**Table S3** Exploratory behaviour induced by KA injection. Mean values of total distance travelled, 95% confidence intervals and respective P values obtained by 1WAY ANOVA with Sidak's multiple comparisons-test at light and dark phases for both genotypes according to each sex and condition (saline and KA).

| **Circadian cycle** | **Genotype** | **Sex** | **Condition** | **N** | **Mean value ± SEM** | **95 % CI** | **P** |
| --- | --- | --- | --- | --- | --- | --- | --- |
| **Light phase** | WT | M | Saline | 10 | 907.1 ± 62.3 | 766.1 – 1048 | **<0.0001** |
|  |  |  | KA | 11 | 323.0 ± 62.1 | 184.7 – 461.3 |  |
|  |  | F | Saline | 12 | 969.7 ± 76.0 | 802.5 - 1137 | **<0.0001** |
|  |  |  | KA | 11 | 444.8 ± 72.41 | 283.5 - 606.1 |  |
|  | *Tsc2^+/-^* | M | Saline | 10 | 969.0 ± 54.6 | 845.6 - 1093 | **<0.0001** |
|  |  |  | KA | 10 | 338.8 ± 82.7 | 151.8 - 525.8 |  |
|  |  | F | Saline | 12 | 1032 ± 72.7 | 872.2 - 1192 | **<0.0001** |
|  |  |  | KA | 11 | 444.8 ± 72.41 | 283.5 – 606.1 |  |
| **Dark phase** | WT | M | Saline | 11 | 1065 ± 72.3 | 904.3 - 1226 | **<0.0001** |
|  |  |  | KA | 12 | 504.6 ± 110.9 | 260.4 - 748.8 |  |
|  |  | F | Saline | 12 | 931.6 ± 84.2 | 746.3 - 1117 | **0.0434** |
|  |  |  | KA | 12 | 414.3 ± 68.6 | 263.3 - 565.3 |  |
|  | *Tsc2^+/-^* | M | Saline | 10 | 1070 ± 68.3 | 919.3 - 1220 | **<0.0001** |
|  |  |  | KA | 12 | 441.1 ± 68.30 | 290.7 + 591.4 |  |
|  |  | F | Saline | 12 | 992.0 ± 67.5 | 843.4 - 1141 | **0.0061** |
|  |  |  | KA | 10 | 411.9 ± 87.6 | 213.7 – 610.2 |  |

CI: Confidence Interval; CM: centimetre; F: Female; KA: Kainic Acid; M: Male; N: Sample size; SEM: Standard Error of the Mean.**Table S4** Anxiety-like behaviour induced by KA injection. Mean values of percentage of time on the centre of the open field, 95% confidence intervals and respective P values obtained by 1WAY ANOVA with Sidak's multiple comparisons-test at light and dark phases for both genotypes according to each sex and condition (saline and KA).

| **Circadian cycle** | **Genotype** | **Sex** | **Condition** | **N** | **Mean value ± SEM** | **95 % CI** | **P** |
| --- | --- | --- | --- | --- | --- | --- | --- |
| **Light Phase** | WT | M | Saline | 12 | 4.8 ± 0.1 | 2.66 – 6.86 | 0.125 |
|  |  |  | KA | 8 | 0.013 ± 0.01 | 0 – 0.03 |  |
|  |  | F | Saline | 12 | 4.7 ± 1.2 | 2.04 – 7.31 | 0.502 |
|  |  |  | KA | 8 | 1.26 ± 0.6 | 0 – 2.72 |  |
|  | *Tsc2^+/-^* | M | Saline | 12 | 9.1 ± 1.9 | 4.85 – 13.35 | **<0.0001** |
|  |  |  | KA | 8 | 0.1 ± 0.1 | 0 – 0.33 |  |
|  |  | F | Saline | 12 | 5.0 ± 0.1 | 2.79 – 7.18 | 0.175 |
|  |  |  | KA | 10 | 0.8 ± 0.4 | 0 – 1.64 |  |
| **Dark phase** | WT | M | Saline | 12 | 8.5 ±2.03 | 4.01 – 12.94 | **0.0067** |
|  |  |  | KA | 11 | 2.4 ± 0.7 | 0.87 – 3.87 |  |
|  |  | F | Saline | 10 | 2.4 ± 0.8 | 0.61 – 4.27 | 0.889 |
|  |  |  | KA | 9 | 0.12 ± 0.09 | 0 – 0.32 |  |
|  | *Tsc2^+/-^* | M | Saline | 12 | 9.7 ± 1.8 | 5.71 – 13.67 | **<0.0001** |
|  |  |  | KA | 9 | 0.8 ± 0.6 | 0 – 2.1 |  |
|  |  | F | Saline | 12 | 7.8 ± 1.8 | 3.8 – 11.85 | 0.1329 |
|  |  |  | KA | 10 | 3.42 ± 1.7 | 0 – 7.27 |  |

CI: Confidence Interval; CM: centimetre; F: Female; KA: Kainic Acid; M: Male; N: Sample size; SEM: Standard Error of the Mean.

**Table S5** Seizure susceptibility induced by KA injection according to the oestrous cycle phase. Mean values of latency times, Racine scores and number of seizures, 95% confidence intervals and respective P values obtained by 3WAY ANOVA with Sidak's multiple comparisons-test at light and dark phase for both genotypes according to each oestrous cycle phase.

|  | **Circadian cycle** | **Genotype** | **Cycle**  **Phase** | **N** | **Mean Value**  **± SEM** | **95 % CI** | **P** |
| --- | --- | --- | --- | --- | --- | --- | --- |
| **Latency Times (s)** | Light phase | WT | O | 7 | 36.7 ± 6.1 | 21.79 - 51.64 | >0.99 |
|  |  |  | P | 4 | 43.3 ± 6.2 | 23.57 - 62.93 |  |
|  |  | *Tsc2^+/-^* | O | 5 | 9.80 ± 1.2 | 6.59 – 13.01 | 0.9976 |
|  |  |  | P | 4 | 23.3 ± 7.1 | 0.88 – 45.62 |  |
|  | Dark phase | WT | O | 8 | 64.4 ± 16.3 | 25.89 – 102.9 | 0.9999 |
|  |  |  | P | 4 | 73.5 ± 6.7 | 52.21 – 94.79 |  |
|  |  | *Tsc2^+/-^* | O | 7 | 21.7 ± 5.1 | 9.31 – 34.12 | >0.99 |
|  |  |  | P | 7 | 27.7 ± 6.2 | 12.45 – 42.98 |  |
| **Racine Scores** | Light phase | WT | O | 6 | 3.5 ± 0.2 | 3.00 – 3.99 | >0.99 |
|  |  |  | P | 5 | 3.7 ± 0.2 | 3.11 – 4.33 |  |
|  |  | *Tsc2^+/-^* | O | 6 | 3.9 ± 0.3 | 3.10 – 4.78 | >0.99 |
|  |  |  | P | 6 | 3.8 ± 0.1 | 3.56 – 4.13 |  |
|  | Dark phase | WT | O | 7 | 3.7 ± 0.2 | 3.26 – 4.18 | **0.0011** |
|  |  |  | P | 4 | 1.5 ± 0.7 | 0.00 – 3.90 |  |
|  |  | *Tsc2^+/-^* | O | 5 | 3.6 ± 0.2 | 3.00 – 4.20 | 0.9943 |
|  |  |  | P | 7 | 4.0 ± 0.4 | 3.081 – 4.95 |  |
| **Number of Seizures** | Light phase | WT | O | 6 | 17.50 ± 0.7 | 15.65 – 19.35 | >0.99 |
|  |  |  | P | 5 | 18.20 ± 1.1 | 15.11 – 21.29 |  |
|  |  | *Tsc2^+/-^* | O | 5 | 19.40 ± 0.7 | 17.52 – 21.28 | >0.99 |
|  |  |  | P | 5 | 20.60 ± 0.4 | 19.49 – 21.71 |  |
|  | Dark phase | WT | O | 8 | 16.0 ± 2.0 | 11.17 – 20.83 | 0.234 |
|  |  |  | P | 4 | 10.0 ± 3.3 | 0.00 – 20.55 |  |
|  |  | *Tsc2^+/-^* | O | 5 | 18.60 ± 1.2 | 15.25 – 21.95 | >0.99 |
|  |  |  | P | 6 | 17.83 ± 1.4 | 14.11 – 21.55 |  |

CI: Confidence Interval; KA: Kainic Acid; N: Sample size; O: Ovulatory phase of oestrous cycle; P: Post-ovulatory phase of oestrous cyle; SEM: Standard Error of the Mean.

**Table S6** Exploratory behaviour induced by KA injection according to the oestrous cycle phase. Mean values of total distance travelled, 95% confidence intervals and respective P values obtained by 1WAY ANOVA with Sidak's multiple comparisons-test at light and dark phases for both genotypes according to each oestrous cycle phase and condition (saline and KA).

| **Circadian cycle** | **Genotype** | **Cycle**  **Phase** | **Condition** | **N** | **Mean value ± SEM** | **95 % CI** | **P** |
| --- | --- | --- | --- | --- | --- | --- | --- |
| **Light phase** | WT | O | Saline | 7 | 925.8 ± 107.9 | 661.7 - 1190 | 0.0871 |
|  |  |  | KA | 6 | 533.7 ± 90.86 | 300.1 – 767.2 |  |
|  |  | P | Saline | 5 | 1031 ± 109.5 | 727.1 - 1335 | **0.0020** |
|  |  |  | KA | 5 | 371.0 ± 94.27 | 109.3 – 632.7 |  |
|  | *Tsc2^+/-^* | O | Saline | 6 | 1042 ± 93.75 | 801.2 - 1283 | 0.1347 |
|  |  |  | KA | 6 | 662.9 ± 183.4 | 191.5 – 1134 |  |
|  |  | P | Saline | 6 | 1022 ± 120.0 | 713.6 - 1330 | **0.0012** |
|  |  |  | KA | 6 | 396.7 ± 101.5 | 135.9 – 657.5 |  |
| **Dark phase** | WT | O | Saline | 8 | 917.2 ± 77.91 | 732.9 - 1101 | **0.0013** |
|  |  |  | KA | 8 | 378.4 ± 100.3 | 141.3 – 615.6 |  |
|  |  | P | Saline | 4 | 960.5 ± 221.9 | 254.2 - 1667 | 0.1189 |
|  |  |  | KA | 4 | 486.0 ± 47.39 | 335.2 – 636.8 |  |
|  | *Tsc2^+/-^* | O | Saline | 5 | 1064 ± 141.9 | 670.4 - 1458 | **0.0423** |
|  |  |  | KA | 5 | 572.5 ± 118 | 245.0 – 900.1 |  |
|  |  | P | Saline | 7 | 940.3 ± 60.63 | 792 - 1089 | **0.0004** |
|  |  |  | KA | 5 | 251.3 ± 87.98 | 7.080 – 495.6 |  |

CI: Confidence Interval; KA: Kainic Acid; N: Sample Size; O: Ovulatory phase of oestrous cycle; P: Post-ovulatory phase of oestrous cycle; SEM: Standard Error of the Mean.

**Table S7** Anxiety-like behaviour induced by KA injection according to the oestrous cycle phase. Mean values of percentage of time in the centre of the open field, 95% confidence intervals and respective P values obtained by 1WAY ANOVA with Sidak's multiple comparisons-test at light and dark phases for both genotypes according to each oestrous cycle phase and condition (saline and KA).

| **Circadian cycle** | **Genotype** | **Cycle**  **Phase** | **Condition** | **N** | **Mean value ± SEM** | **95 % CI** | **P** |
| --- | --- | --- | --- | --- | --- | --- | --- |
| **Light phase** | WT | O | Saline | 7 | 4.059 ± 1.3 | 0.77-7.35 | 0.8350 |
|  |  |  | KA | 5 | 1.2 ± 0.7 | 0 - 2.96 |  |
|  |  | P | Saline | 5 | 5.5 ± 2.3 | 0 - 11.95 | >0.99 |
|  |  |  | KA | 4 | 6.1 ± 4.8 | 0 - 21.31 |  |
|  | *Tsc2^+/-^* | O | Saline | 6 | 4.3 ± 1.0 | 1.77 - 6.78 | 0.728 |
|  |  |  | KA | 5 | 0.9 ± 0.37 | 0 – 1.9 |  |
|  |  | P | Saline | 6 | 5.7 ± 1.8 | 1.09 - 10.31 | 0.2522 |
|  |  |  | KA | 5 | 0.7 ± 0.7 | 0 - 2.66 |  |
| **Dark phase** | WT | O | Saline | 7 | 1.8 ± 0.7 | 0.14 - 3.45 | 0.9846 |
|  |  |  | KA | 6 | 0.01 ± 0.005 | 0 - 0.02 |  |
|  |  | P | Saline | 4 | 6.4 ± 3.0 | 0 - 15.97 | 0.289 |
|  |  |  | KA | 4 | 0.8 ± 0.5 | 0 – 2.451 |  |
|  | *Tsc2^+/-^* | O | Saline | 4 | 2.7 ± 0.9 | 0 - 5.55 | >0.99 |
|  |  |  | KA | 5 | 3.497 ± 2.2 | 0 – 9.62 |  |
|  |  | P | Saline | 7 | 9.1 ± 2.1 | 3.99 -14.21 | **0.0020** |
|  |  |  | KA | 5 | 0.4 ± 0.3 | 0 - 1.19 |  |

CI: Confidence Interval; KA: Kainic Acid; N: Sample Size; O: Ovulatory phase of oestrous cycle; P: Post-ovulatory phase of oestrous cycle; SEM: Standard Error of the Mean.
